# Heterogeneous Activation of NaClO by Nano-CoMn_2_O_4_ Spinel for Methylene Blue Decolorization

**DOI:** 10.3390/ijms26030940

**Published:** 2025-01-23

**Authors:** Tongwen Zhao, Gang Han, Juan Bai, Xiaogang Wu

**Affiliations:** School of Urban Construction, Yangtze University, Jingzhou 434023, China; hhuhjxyztw@163.com (T.Z.); hh123gang@163.com (G.H.); 2023710765@yangtzeu.edu.cn (J.B.)

**Keywords:** nanomaterial, spinel, sodium hypochlorite, superoxide radical, organic pollutant, advanced oxidation process

## Abstract

In this study, the nano-spinel CoMn_2_O_4_ was synthesized by coprecipitation pyrolysis and employed to heterogeneously activate hypochlorite (NaClO) for the oxidative decolorization of methylene blue (MB). The crystal structure, elemental composition, surface morphology, and microstructure of the prepared CoMn_2_O_4_ nano-spinel were analyzed using a series of characterization techniques. The pyrolysis temperature was screened on the basis of MB decolorization efficiency and the leaching of metal ions during the reaction. The MB decolorization efficiency was compared using different catalysts and process. The impacts of CoMn_2_O_4_ dosage, effective chlorine dose, MB concentration, and initial pH on MB decolorization were explored. The catalytic mechanism of MB oxidation was elucidated through quenching experiments combined with radical identification. The degradation pathway of MB was preliminarily proposed based on the detection of the intermediates. The reusability of recycled CoMn_2_O_4_ was finally investigated. The results revealed that maximal MB oxidation efficiency and minimal leaching of Co and Mn ions were achieved at the calcination temperature of 600 °C. Complete oxidative decolorization of MB within 40 min was obtained at an initial MB concentration of 50 mg/L, a CoMn_2_O_4_ dosage of 1 g/L, an effective chlorine dose of 0.1%, and an initial pH of 4.3. Superoxide radical (O_2_^•−^) was found to be dominantly responsible for MB decolorization according to the results of radical scavenging experiments and electron paramagnetic resonance. The CoMn_2_O_4_ spinel can be recycled for five cycles with the MB removal in the range of 90.6~98.7%.

## 1. Introduction

Fast-paced industrial advancement has significantly eased human activities and daily life. Nevertheless, the emergence of novel wastewater types due to industrial development has presented formidable challenges to conventional water treatment methodologies. These traditional techniques frequently prove ineffective in managing emerging pollutants and fall short of meeting the increasingly stringent discharge standards [1]. In recent years, environmental scientists have carried out comprehensive research and discovered that among the array of pollution remediation technologies (biological, physical, chemical, etc.), advanced oxidation processes (AOPs) hold the greatest potential, demonstrating high efficiency in treating recalcitrant or difficult-to-mineralize organic pollutants [2,3,4,5]. In homogeneous/heterogeneous AOP reactions, highly oxidative species (such as HO^•^, SO_4_^•−^, O_2_^•−^, ^1^O_2_) attack target pollutants, mineralizing them into small organic molecules and even carbon dioxide and water. AOPs provide advantages in terms of treatment speed, efficiency, and environmental friendliness [6,7]. To date, various AOP technologies have received widespread attention, including Fenton oxidation [8,9], photocatalytic oxidation [10], ozone oxidation [11], and persulfate oxidation [12]. However, homogeneous AOPs have limitations, such as poor catalyst availability and recovery difficulties. Some of these AOP technologies are costly (e.g., high energy input, expensive chemicals), which limits their large-scale application [7].

Considering the limitations of existing AOP technologies, an increasing number of researchers are now directing their attention towards the utilization of sodium hypochlorite (NaClO) for wastewater treatment. NaClO, as an oxidant, can also generate nascent oxygen species, which exhibit similar decomposition characteristics to those of H_2_O_2_ [13]. NaClO has several notable advantages, including a broader pH range of applicability compared to the Fenton process, greater cost-effectiveness in manufacture than alternative oxidants (with 10% NaClO priced at 82.8 $/t, in contrast to 30% H_2_O_2_ at 164.4 $/t and persulfate at 931.6 $/t as of 8 January 2025), and potent disinfection properties [14,15]. Furthermore, the waste produced after NaClO undergoes oxidation is non-toxic sodium chloride (NaCl), which has little effect on the environment at low concentrations [16]. Given these characteristics, the oxidative strength and decolorization efficacy using NaClO in treating wastewater are superior to conventional oxidants, demonstrating significant potential for practical applications. Stephanie et al. demonstrated the oxidation potential of hypochlorous acid in promoting substitution reactions [17]. Existing research indicated that chlorine addition and coupling reactions were the main mechanisms of oxidative degradation of organic pollutants using NaClO [18,19]. Therefore, direct use of NaClO to oxidize pollutants may generate organic chlorides, which could be more harmful than the original pollutants [20]. However, this issue can be addressed by combining NaClO with other processes [18].

Studies have shown that certain metal-based catalysts can effectively enhance the oxidation capacity of sodium hypochlorite (NaClO), thereby improving its oxidative performance [13]. Transition metals (such as manganese, cobalt, iron, nickel, and copper) are easily oxidized by NaClO to higher valence states due to the instability of their valence electrons, gaining strong oxidation capabilities. Thus, they can serve as effective catalysts to promote redox reactions [21]. Meanwhile, some metal-containing oxides are magnetic (such as iron, cobalt, nickel, manganese), which can aid their recovery after water treatment. The oxidative nature of NaClO is attributed to the O-Cl bond, which is easily broken at room temperature [22]. Under the action of transition metal catalysts, the broken O-Cl bond may lead to the production of different states of chlorine and reactive oxygen species, further promoting oxidation reactions (Equation (1)) [23].

(1) $$2\;ClO^{-}\overset{\mathrm{metals}}{\to }\;2Cl^{-}+O_{2}$$

Based on this characteristic, the active species produced by transition-metal-catalyzed NaClO are capable for decomposition in wastewater. Li et al. [24] demonstrated that the ferrate (VI)–hypochlorite liquid mixture maintained a high decolorization efficiency for initial 10 mg/L Orange II within a broad pH range of 3.0 to 11.0. Meanwhile, Fu et al. [25] achieved a near-100% decolorization rate when treating simulated wastewater containing an initial 400 mg/L Reactive Red concentration 195 using a combination of zero-valent iron/activated carbon, microwave discharge electrodeless lamp, and sodium hypochlorite (ZVI/AC-MDEL/NaClO). In recent years, Mn- and Co-based catalysts have received widespread attention [26]. Cobalt oxides stand out for their abundant oxygen vacancies and oxygen storage capacity. The presence of abundant Co^2+^ indicates the generation of oxygen vacancies in the catalyst, which is beneficial for promoting catalytic activity [27]. The cobalt oxide catalyst immobilized on graphene oxide (GO) was able to activate peroxymonosulfate (PMS), resulting in the complete degradation of 0.2 mM Orange II in water within 6 min [28]. Manganese oxides are widely used in catalysts due to their multiple oxidation states (Mn^4+^, Mn^3+^, Mn^2+^). According to the Mars-van Kreveen model, a large amount of Mn^4+^ is beneficial for increasing the ratio of lattice oxygen (O_latt_) to adsorbed oxygen (O_ads_), with lattice oxygen playing a positive role in enhancing catalytic activity [29,30]. Zhi et al. [13] prepared MnO_2_–mullite–cordierite composite particles and utilized NaClO as the oxidant for the decolorization of methylene blue (MB). The experimental results indicated that this system could achieve a near-100% decolorization rate for 100 mg/L MB.

Nano-spinel (XY_2_O_4_, X/Y are transition metal ions) catalysts have attracted widespread attention due to the excellent synergistic effect between the two metal ions and the low leaching of metal ions in aqueous solutions [31,32]. Among them, CoMn_2_O_4_ has been extensively studied due to its structural stability, low cost, environmental friendliness, and excellent catalytic activity. Nguyen et al. [33] reported a three-dimensional flower-like CoMn_2_O_4_ catalyst with high-performance in Fenton-like process. With the synergistic effect of Co and Mn, abundant oxygen vacancies were generated, accelerating electron transfer and greatly improving the catalytic performance for tetracycline degradation. Xu et al. [34] revealed a novel carbon-doped CoMn_2_O_4_/Mn_3_O_4_ composite in activating PMS for ciprofloxacin degradation, exhibiting excellent catalytic performance and good recyclability. Yang et al. [35] reported an efficient catalyst with CoMn_2_O_4_ uniformly dispersing on the surface of halloysite to expose more active sites. Nevertheless, it has rarely been reported that a heterogeneous activation of NaClO by the nano-CoMn_2_O_4_ spinel for the decomposition of organic pollutants though CoMn_2_O_4_ has been employed in wastewater treatment.

Synthetic dyes are widely used in various industries, such as textiles, cosmetics, printing, pharmaceuticals, and food processing, with approximately 7 × 10^5^ tons of different types of dyes and pigments being produced globally each year [36]. Among these, azo dyes, which have an -N=N- unit in their molecular structure, account for 60–70% of all synthetic dyes produced [37]. It is reported that the textile industry alone consumes over 10 billion liters of water and 800,000 tons of dyes daily, with 120,000 tons ultimately entering wastewater [38]. Azo dyes and their decomposition products are highly toxic due to their carcinogenic, teratogenic, and mutagenic properties, which can not only degrade water quality but also cause adverse reactions, such as genetic mutations, skin inflammation, allergies, and skin irritation, when humans are exposed to synthetic azo dyes [39]. Methylene blue (MB), a heterocyclic aromatic azo dye, is a highly carcinogenic thiazine contaminant and one of the most widely used dyes in various industries [40]. MB has a complex structure and is resistant to biodegradation, posing potential harm to ecosystems and being difficult to decolorize [41,42]. Once ingested by humans, it can cause various injuries, including to the nervous system and eyes [36].

Herein, in this work, we systematically investigated the decolorization of methylene blue (MB) by the CoMn_2_O_4_-activated NaClO process. The performance of MB decolorization and impacted parameters in the CoMn_2_O_4_/NaClO system were investigated. The primary active species for MB oxidation was elucidated. Finally, possible intermediates during MB degradation process were identified, and a potential degradation pathway for MB was proposed.

## 2. Results and Discussion

### 2.1. Characterization of CoMn_2_O_4_

**Figure 1:**
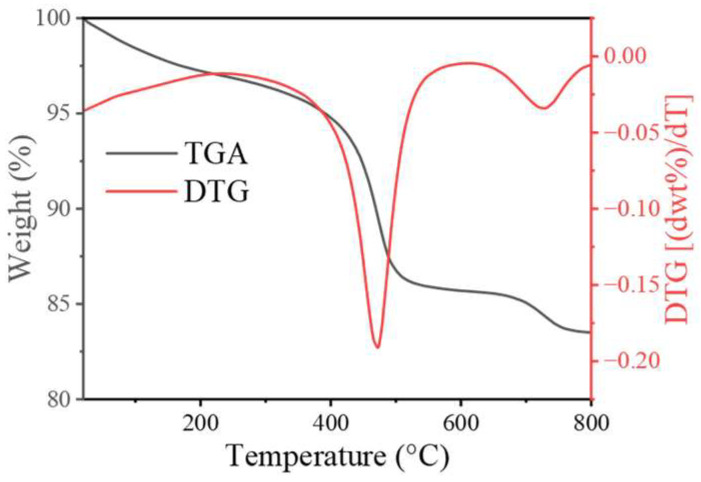
TGA (black plot) and DTG (red plot) analysis of as-prepared CoMn_2_O_4_.

Thermogravimetric analysis was carried out from room temperature to 800 °C in a static air atmosphere to evaluate the thermal properties of the catalyst (Figure 1). The first mass loss was about 6.0% below 400 °C, which resulted from the evaporation of anhydrous ethanol, crystal water in the metallic precursor, and a little water [43]. The second mass loss was 8.0% (400–500 °C), which might be related to the conversion of inorganic cobalt to organic cobalt [44] and inorganic manganese to organic manganese [45]. The third mass loss was 2.5% (685–800 °C), which was associated with the phase distortion of organic cobalt and organic manganese due to the gradual loss of excess oxygen [45,46]. Therefore, an annealing temperature of 600 °C was employed.

**Figure 2:**
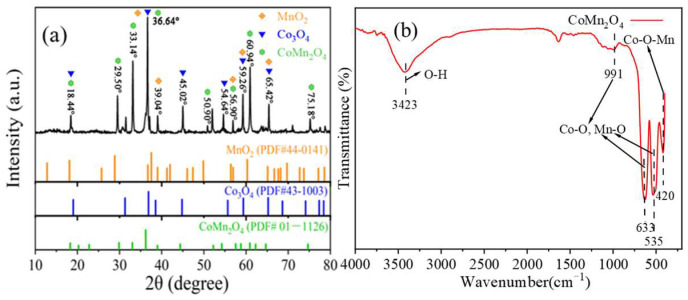
(**a**) The XRD pattern of CoMn_2_O_4_; (**b**) FT-IR spectrum of CoMn_2_O_4_.

To verify against the pure CoMn_2_O_4_ spinel, the prepared catalyst was characterized using XRD. As shown in Figure 2a, the diffraction peaks at 18.44°, 29.50°, 33.14°, 36.64°, 50.90°, 56.90°, 60.94°, and 75.18° of the prepared materials agreed well with the peaks of the pure CoMn_2_O_4_ according to the standard card CoMn_2_O_4_ (PDF#44-0141), indicating that the CoMn_2_O_4_ spinel was successfully synthesized [47]. The diffraction peaks at 2θ = 18.44°, 36.64°, 45.02°, 54.64°, 59.26°, and 65.42° fitted well with Co_3_O_4_. At the same time, the diffraction peaks of MnO_2_ at 2θ were 36.64°, 39.04°, 56.90°, 59.26°, and 65.42°, respectively [33]. At the degrees of 29.86°, 33.02°, 36.20°, and 62.26°, the materials exhibited high diffraction intensity and sharp shape, implying the high crystallinity of the catalysts and excellent arrangement of internal particles [48].

Figure 2b presents the FT-IR spectrum of the CoMn_2_O_4_ spinel. The peak at 3423 cm^−1^ can be attributed to the O-H stretching vibration of associated OH groups on the surface of the catalyst [49]. The peaks at 991, 633, and 533 cm^−1^ were attributed to the lattice vibration of cobalt-based spinel and the stretching vibrations of Co-O and Mn-O, respectively [50,51]. The peak at 430 cm^−1^ can be referred to the characteristic vibration of the Co-O-Mn bond [52]. These peaks confirmed the formation of spinel CoMn_2_O_4_ after calcination.

**Figure 3:**
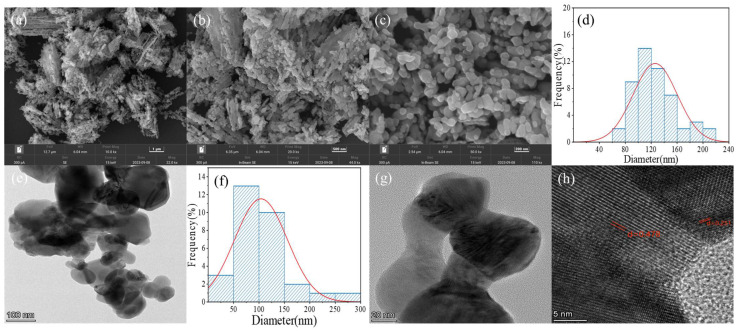
The SEM images of CoMn_2_O_4_ samples (**a**–**c**), the particle size distribution histogram of SEM (**d**), the TEM images of CoMn_2_O_4_ samples (**e**,**g**,**h**), the particle size distribution histogram of TEM (**f**).

Figure 3a–d depict the SEM images of the CoMn_2_O_4_ spinel at different magnifications and the particle size distribution histogram of SEM. Figure 3a revealed that the morphology of the CoMn_2_O_4_ spinel consists of layered particles with a rough surface, which potentially increase its specific surface areas and the active sites between pollutant molecules and the catalysts, facilitating the catalytic reactions [48]. Figure 3b–d demonstrate that the CoMn_2_O_4_ catalyst particles were uniformly distributed with a loose and rough structure, whose average particle size was found to be 125 nm (approx.) from the particle size distribution histogram, exhibiting a porous structure that can provide more extensive active reaction sites [53,54].

The TEM images and the particle size distribution histogram of the TEM of CoMn_2_O_4_ are presented in Figure 3e–h. Figure 3e–g illustrated that CoMn_2_O_4_ was composed of layered nanosheets, whose average particle size was found to be 105 nm (approx.) from the particle size distribution histogram, whose result was similar to SEM. In Figure 3h, abundant lattice fringes were observed, with lattice spacings of d = 0.251 nm corresponding to the (311) plane of CoMn_2_O_4_, and d = 0.478 nm related to the (111) plane of CoMn_2_O_4_.

**Figure 4:**
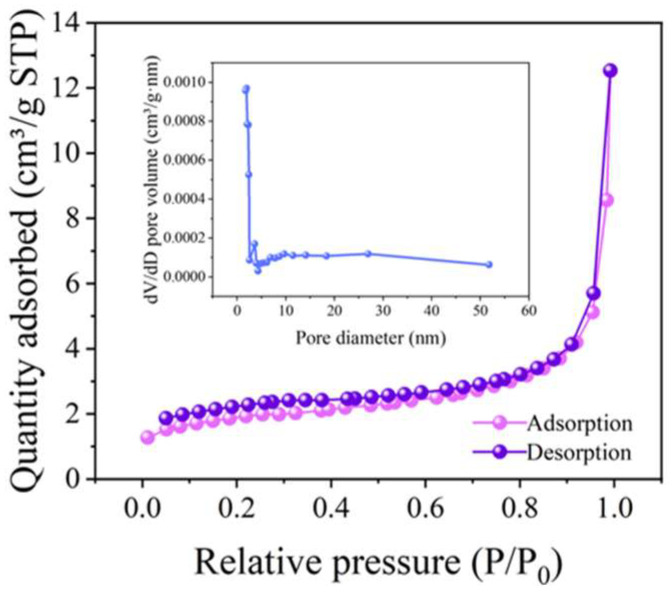
N_2_ adsorption–desorption isotherms of CoMn_2_O_4_. (insert: pore size distribution).

The specific surface area and pore size distribution were obtained through N_2_ adsorption–desorption isotherms by Bruner–Emmett–Teller (Figure 4). The nitrogen physisorption analysis revealed that the specific surface area of CoMn_2_O_4_ was about 6.7865 m^2^/g, with a pore volume of 0.01939 cm^3^/g. CoMn_2_O_4_ showed typical type-IV isotherms with hysteresis loops. The hysteresis was suggestive of capillary condensation [55], indicating they were mesoporous materials (2–50 nm) and the size of these pores to be about 19.7 nm. The electrochemical property of spinel is stable in an aqueous environment and under illumination with a mechanism conduction by electron hopping [56]. Thus, based on the BET analysis, the quantity and capability of mesoporous CoMn_2_O_4_ were able to accommodate some molecular magnitude and broaden pathways for ion transport [55]. The mesoporous properties and surface area could provide CoMn_2_O_4_ with available active sites, which could provide opportunities for the catalyst to be in full contact with oxygen and electrolyte, contributing to its enhanced catalytic activities and the desorption of products to accelerate the reaction rate [57,58].

**Figure 5:**
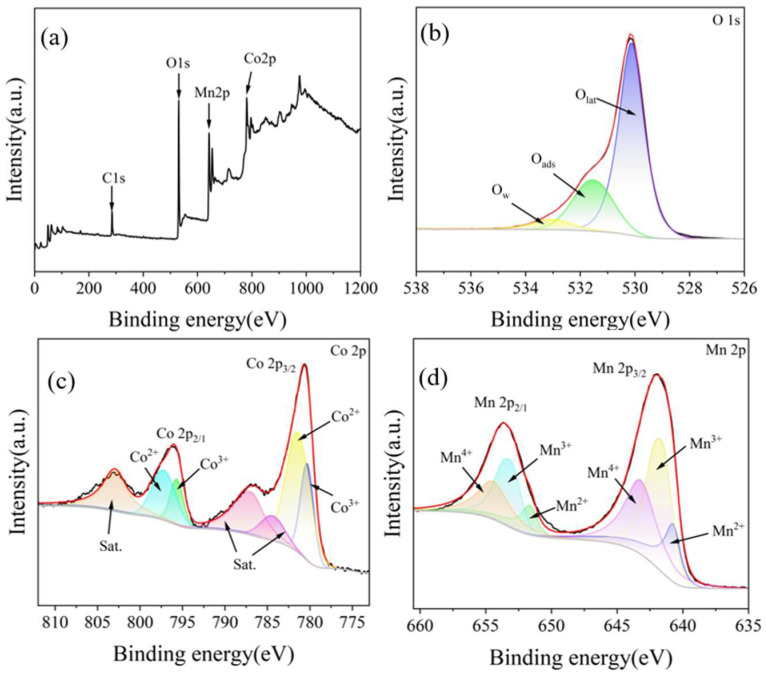
XPS spectra of CoMn_2_O_4_ samples in different reaction stages: (**a**) survey scan; (**b**) O 1s; and (**c**) Co 2p and (**d**) Mn 2p.

XPS is the primary analysis of the elemental composition and valence states on the surface of the materials. Figure 5 depicts the XPS spectra of the prepared CoMn_2_O_4_ spinel. Figure 5a displays the full XPS spectrum of the CoMn_2_O_4_ catalyst, revealing only Mn, Co, and O elements in the prepared materials with no observed impurities other than carbon. This result further confirmed that the product calcinated at 600 °C was pure CoMn_2_O_4_.

In Figure 5b, the O 1s spectrum can be deconvoluted into three peaks. The peaks centered at 530.12 eV, 531.53 eV, and 533.05 eV were identified as O_latt_ (lattice oxygen), O_ads_ (adsorbed oxygen), and O_sur_ (surface adsorbed OH groups or molecular water) [34,59]. O_latt_ was the main oxygen species in the CoMn_2_O_4_ sample, which was due to the formation of the Co-Mn solid solution, leading to lattice defects, exposing more active sites, and increasing the concentration of lattice oxygen. The increase in the lattice oxygen content contributed to the catalytic activity of the sample [60]. Furthermore, the atomic ratio of O_latt_/O_ads_ affected the catalytic oxidation of organic pollutants. The O_latt_/O_ads_ atomic ratio of the CoMn_2_O_4_ nano-spinel was as high as 2.69, indicating high catalytic oxidation performance [59].

In the high-resolution Co 2p spectrum in Figure 5c, the Co 2p spectrum was fitted with a pair of spin-orbit peaks (Co 2p_3/2_ and Co 2p_1/2_). Peaks corresponding to Co^3+^ at the Co 2p_3/2_ and Co 2p_1/2_ energy levels were observed at 780.34 eV and 794.67 eV, respectively. Additionally, peaks associated with Co^2+^ at the Co 2p_3/2_ and Co 2p_1/2_ energy levels were identified at 781.42 eV and 797.21 eV, respectively. The findings indicated that Co exists in mixed valence states of Co^3+^ and Co^2+^ [60]. The presence of low-valence Co^2+^ may indicate the generation of oxygen vacancies in the nanomaterials, which was beneficial for promoting catalytic activity [48]. The peaks located at 784.39 eV, 786.93 eV, and 802.94 eV were attributed to satellite peaks [61].

As displayed in Figure 5d, the high-resolution Mn 2p spectrum was also fitted with a pair of spin-orbit peaks (Mn 2p_3/2_ and Mn 2p_1/2_). The peaks at 640.74 eV, 641.63 eV, and 643.22 eV corresponding to Mn 2p_3/2_ were assigned to Mn^2+^, Mn^3+^, and Mn^4+^, respectively. Furthermore, the peak of Mn 2p_3/2_ could be deconvoluted into three sub-peaks at 652.64 eV, 653.17 eV, and 654.44 eV, which were also attributed to Mn^2+^, Mn^3+^, and Mn^4+^, respectively [48,62]. It has been reported that redox reactions produced oxygen vacancies, and a higher concentration of Mn^3+^ was considered to indicate the presence of more oxygen vacancies [48,63].

### 2.2. Catalytic Performance of CoMn_2_O_4_

**Figure 6:**
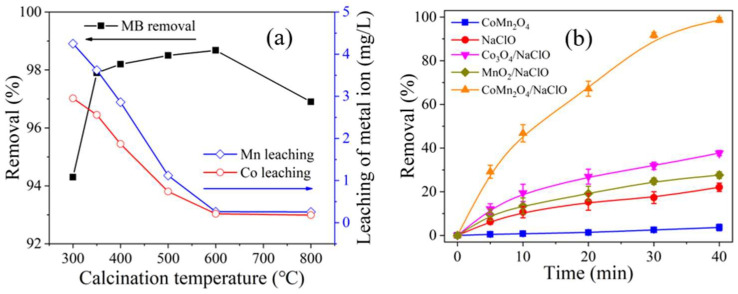
(**a**) Effects of CoMn_2_O_4_ calcination temperature on the decolorization efficiency of MB and the leaching of metal ions; (**b**) MB degradation under different systems. Conditions: [MB] = 10 mg/L, [Catalysts] = 1 g/L, effective chlorine 0.05%, pH = 4.3 ± 0.1.

The calcination temperature not only affected the structural properties of the nanomaterials, but also influenced the stability of the catalysts. Figure 6a illustrates the impact of calcination temperature on MB decolorization and the leaching of metal ions from the nano-spinel. As the calcinating temperature increased from 300 °C to 600 °C, the MB decolorization rate increased from 94.3% to 98.7%. When the temperature further increased to 800 °C, the degradation rate was 96.9%. This is because at lower temperatures, Co and Mn salts cannot fully decompose into their respective oxides, resulting in lower catalyst purity and poorer degradation performance. At higher temperatures, the bonding state of metal oxides in the catalyst may change, leading to loss of activity.

The leaching of Mn and Co decreased from 4.25 mg/L and 2.95 mg/L to 0.25 mg/L and 0.18 mg/L, respectively, as the calcination temperature increased from 300 °C to 800 °C. Higher calcination temperatures better remove water and crystallization reactions in the catalyst, producing more stable metal oxides and reducing heavy metal leaching. Considering these factors, 600 °C was chosen as the optimal calcination temperature.

Figure 6b depicts the MB decolorization efficiency in different systems. When only the CoMn_2_O_4_ nano-catalyst was added, the MB decolorization rate was merely 3.7%, indicating weak adsorption of MB by CoMn_2_O_4_, consistent with the findings of Guo, Li, and Fan [53,62,64]. When NaClO was used alone, the MB decolorization efficiency was 22.1%, which can be ascribed to the strong oxidative properties of NaClO [65]. With the involvement of Co_3_O_4_ and MnO_2_, the decolorization of MB was both promoted with efficiencies of 37.7% and 27.6%, respectively, which was due to the activation of NaClO by metal oxides [13]. The CoMn_2_O_4_/NaClO system exhibited greatest catalytic activity, with an MB decolorization rate of 98.7%, demonstrating that the addition of the CoMn_2_O_4_ enhanced NaClO oxidation. This is attributed to the synergistic effect between metallic compositions in the nano-spinel and ClO^−^ species [13]. In Table 1, the previous studies on the degradation of methylene blue by the corresponding catalyst or sodium hypochlorite were provided. It is also proved that the spinel catalyst CoMn_2_O_4_ has the ability to rapidly degrade methylene blue in a low concentration of NaClO environment at a lower dose.

### 2.3. Effect of Parameters

**Figure 7:**
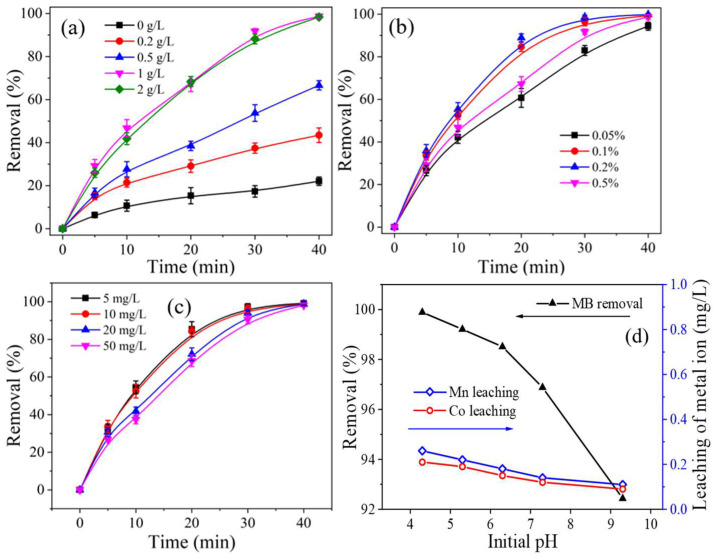
Effects of (**a**) CoMn_2_O_4_ dosage, conditions: [MB] = 10 mg/L, effective chlorine 0.05%, pH = 4.3 ± 0.1; (**b**) effective chlorine concentration, conditions: [MB] = 10 mg/L, [CoMn_2_O_4_] = 1 g/L, pH = 4.3 ± 0.1; (**c**) MB concentration, conditions: [CoMn_2_O_4_] = 1 g/L, pH = 4.3 ± 0.1; (**d**) initial pH and the leaching of metal ions, conditions: [MB] = 50 mg/L, effective chlorine 0.1%, [CoMn_2_O_4_] = 1 g/L.

Figure 7a illustrates the effect of CoMn_2_O_4_ dosage (0–2 g/L) on MB decolorization efficiency. As the CoMn_2_O_4_ dosage increased from 0 g/L to 1 g/L, the MB degradation rate increased from 43.8% to 98.67%. This significant improvement may be due to the increased catalytic metal compositions and growing number of active sites with the increasement of nano-spinel [53]. Further increasing the nano-spinel dosage to 2 g/L did not provide additional benefits. Thus, 1 g/L was selected as the optimal catalyst dosage, which is significantly lower than those reported in previous studies [13,53], potentially reducing large-scale application costs [15].

Figure 7b shows the effect of the initial effective chlorine concentration on MB degradation efficiency. As the effective chlorine concentration increased from 0.05% to 0.2%, the MB decolorization efficiency improved significantly due to enhanced active oxygen and hypochlorite content [53,67]. In addition, with higher NaClO, the amount favors NaClO molecules into the active sites, thus producing more active oxidative species [68]. However, when the concentration increased to 0.5%, the decolorization efficiency decreased dramatically. This may be due to (1) a scavenging effect similar to H_2_O_2_ scavenging hydroxyl radicals [13,69] and (2) slower NaClO decomposition caused by high pH environment in the presence of NaOH in commercial NaClO.

Figure 7c demonstrates the MB decolorization efficiency at different initial MB concentrations (5–50 mg/L). Although the degradation rate decreased slightly with increasing MB concentration, the degradation efficiency remained above 98.5% after 40 min for all concentrations. This indicates that the CoMn_2_O_4_/NaClO system can achieve deep decolorization of MB even at high substrate concentrations. Therefore, MB of 50 mg/L was used in the subsequent experiments.

Figure 7d depicts the impact of initial pH (4.3–9.3) on MB degradation. As pH increased from 4.3 to 9.3, the MB decolorization rate ranged narrowly around 95.0%, revealing the good adaptability to pH of the CoMn_2_O_4_/NaClO process. The leaching of Mn and Co decreased from 0.26 mg/L and 0.21 mg/L to 0.11 mg/L and 0.09 mg/L, respectively, as the initial pH increased from 4.3 to 9.3. Although the metal precipitation rate was positively correlated with the degradation efficiency, the initial pH value had little effect on the degradation efficiency of MB. This probably can be attributed to the findings that the pH value of the solution maintained at around 10.0 after addition of NaClO, and the pH values varied narrowly during the reactions, although the initial pH value ranged in the range of 4.3–9.3. Guo reported that MB oxidation was accelerated at a lower pH circumstance in the Ni-Fe bimetallic catalyst/NaClO system, which was explained by the stronger oxidative capacity of HOCl derived from NaClO under acidic conditions [53]. This finding in the current study implied that the dominant active species and mechanism for MB oxidation probably differed from Guo’s study, the species corresponded to HOCl (e.g., ^1^O_2_, ClO^•^), and acidic-induced species probably were not responsible for MB oxidation in this case.

**Figure 8:**
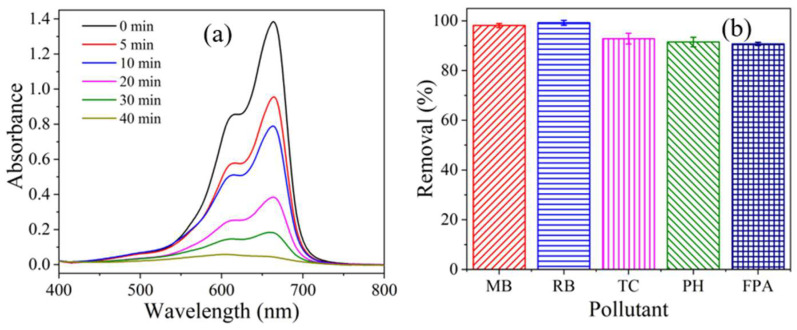
(**a**) The spectral evolution of MB and (**b**) oxidative performance of various ubiquitous pollutants by CoMn_2_O_4_/NaClO system under the optimal conditions.

Finally, complete oxidative decolorization of MB within 40 min was obtained at an initial MB concentration of 50 mg/L, CoMn_2_O_4_ dosage of 1 g/L, effective chlorine dose of 0.1%, and initial pH of 4.3. Under these circumstances, the spectral evolution of MB during the reaction was recorded in Figure 8a. The significant decrease in the characteristic absorption band intensity (λmax = 662 nm) and a shift over time indicated that MB underwent deep decolorization and the molecular structure of MB was destroyed.

Oxidative performance of various ubiquitous pollutants by the CoMn_2_O_4_/NaClO system under the optimal conditions was further explored, and the results were shown in Figure 8b. It can be seen that removals of 99.2%, 92.8%, 91.5%, and 90.7% were achieved within 40 min for Rhodamine B (RB), tetracycline hydrochloride (TC), phenol (PH), and norfloxacin (FPA), respectively, revealing the remarkable oxidation capacity of the CoMn_2_O_4_/NaClO process.

### 2.4. Mechanism Analysis

**Figure 9:**
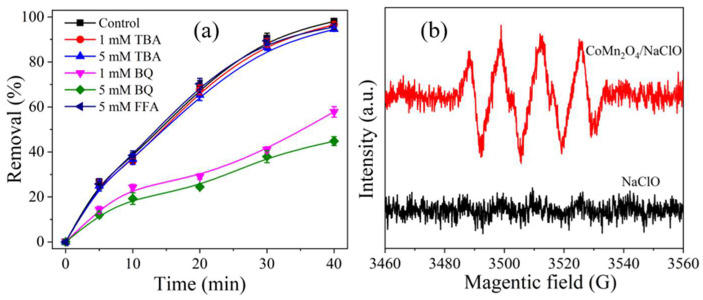
(**a**) The spectral evolution of MB under the system of CoMn_2_O_4_/NaClO; (**b**) degradation of various pollutants by CoMn_2_O_4_/NaClO. Conditions: [pollutant] = 50 mg/L, [Catalysts] = 1 g/L, effective chlorine 0.1%, pH = 4.3 ± 0.1.

It has been reported that in NaClO-involved heterogeneous AOPs, active species such as O_2_^•−^, HO^•^, and ^1^O_2_ were inducible and predominant for decomposition of organic contaminants. To identify the main active species, the commonly recognized radical quenchers tert-butanol (TBA), benzoquinone (BQ), and furfuryl alcohol (FFA) were employed for capturing HO^•^, O_2_^•−^, and ^1^O_2_, respectively [53,70,71]. As shown in Figure 9a, the addition of either 1 mM or 5 mM TBA was only slightly inhibited by the decolorization of MB with the efficiencies reduced from 98.1% to 96.6% and 94.5%, respectively. However, the addition of benzoquinone played a prominent inhibitory effect on MB decolorization, reducing the removal of MB from 98.1% to 57.8% with 1 mM BQ and 44.8% with 5 mM BQ. The results indicated that HO^•^ did not contribute to MB decolorization; however, O_2_^•−^ or O_2_^•−^ derived ^1^O_2_ (Equation (2)) [72] probably was responsible for MB decolorization.

O_2_^•−^ + HO^•^ → ^1^O_2_ + OH, (2)

To identify the contribution of ^1^O_2_, 5 mM FFA was introduced to the CoMn_2_O_4_/NaClO system. Interestingly, the decolorization of MB barely diminished (95.6%), implying the negligible contribution of ^1^O_2_ to MB removal. The confirmation of the neglected contribution of ^1^O_2_ verified the deduction that acidic-induced species (e.g., HOCl, ^1^O_2_, ClO^•^) were not contributive to MB removal, as discussed in the section on the impact of the initial pH. To further confirm the presence of O_2_^•−^ in the CoMn_2_O_4_/NaClO system, an electron paramagnetic resonance (EPR) analysis was conducted, and the results were shown in Figure 9b. No obvious signal was found by the addition of NaClO alone, and after the addition of both NaClO and CoMn_2_O_4_, the typical sextuple EPR spectrum corresponding to O_2_^•−^ was observed, indicating that O_2_^•−^ was produced in the CoMn_2_O_4_/NaClO system [65,73].

Mn^4+^/Mn^3+^ + ClO^−^ → Mn^3+^/Mn^2+^ + O_2_^•−^ + Cl^−^, (3)

Co^3+^ + ClO^−^ → Co^2+^ + O_2_^•−^ + Cl^−^, (4)

O_2_ + e^−^ → O_2_^•−^, (5)

Based on the above findings, the possible decolorization mechanism of MB in the CoMn_2_O_4_/NaClO system is proposed. Mn^4+^, Mn^3+^, and Co^3+^ from the nano-spinel CoMn_2_O_4_ may activate NaClO to directly produce O_2_^•−^ for catalytic decomposition of MB (Equations (3) and (4) [74,75,76]. Additionally, the small amount of generated O_2_ via Equation (1) could capture electrons donated by oxygen vacancies to form O_2_^•−^ (Equation (5)). Finally, MB molecules were attacked, followed by the breakage of structural bonds to form intermediate products, and then were mineralized into molecules of H_2_O and CO_2_ [13].

### 2.5. Proposed MB Decolorization Pathway

**Figure 10:**
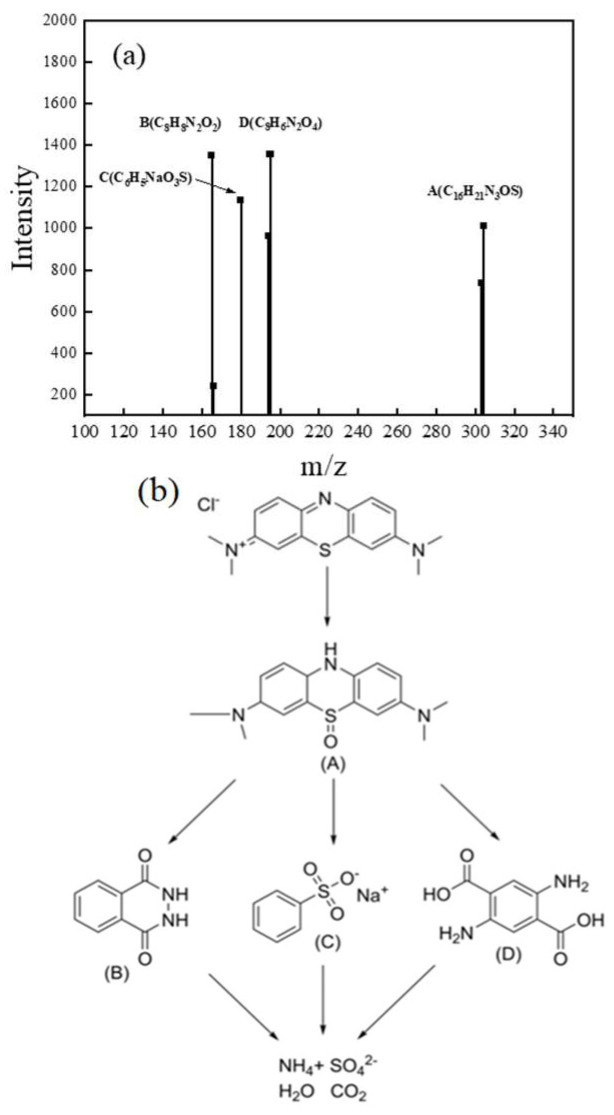
(**a**) The mass spectra of MB solution after reaction with CoMn_2_O_4_/NaClO; (**b**) the proposed pathway of MB decolorization.

High-Performance Liquid Chromatography-Mass Spectrometry (HPLC-MS) analyses were used to identify intermediates formed during MB decolorization. As illustrated in Figure 10a, products of A (C_16_H_21_N_3_OS), B (C_8_H_8_N_2_O_2_), C (C_6_H_5_NaO_3_S), and D (C_8_H_6_N_2_O_4_) were presented in the full-scan MS spectra. According to these detected intermediates, Figure 10b presents possible degradation pathways for MB. The initial oxidation occurred at the sulfur site, producing intermediate A [77]. Product A was further oxidized to generate intermediates B, C, and D in the sequential reaction. The dimethylamino group in MB undergone progressive transformation to methyl, aldehyde, and nitro groups, before further mineralization to ammonium (NH_4_^+^), sulfate (SO_4_^2−^), H_2_O, and CO_2_. Some studies reported some small organic acid products during the degradation process [78], which was not detected in this study, probably due to the fact that the primary products had not been mineralized to this extent within limited reaction time (10 min) of the sample. The cracking mode and the product situation of the benzene ring in the late degradation stage of MB should be further explored.

### 2.6. The Reusability of CoMn_2_O_4_

**Figure 11:**
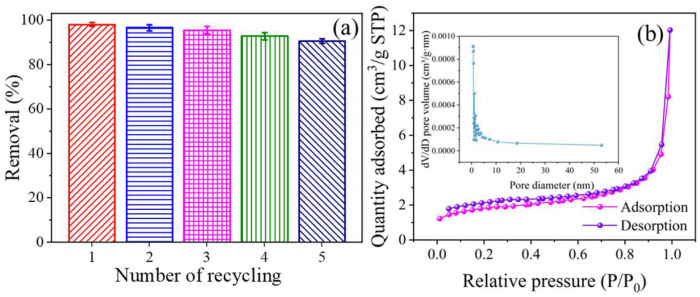
(**a**) Removal of MB using the recycled CoMn_2_O_4_. Conditions: [MB] = 50 mg/L, [CoMn_2_O_4_] = 1 g/L, effective chlorine 0.1%, pH = 4.3 ± 0.1; (**b**) N_2_ adsorption–desorption isotherms of CoMn_2_O_4_ after repeat discoloration experiment (insert: pore size distribution).

The reusability of nano-CoMn_2_O_4_ was further investigated as illustrated in Figure 11a. The nano-spinel maintained high catalytic activity even after five cycles of use, achieving over 90% MB decolorization rate. This result was consistent with the findings of Guo and Zhang [53,79]. The slight decrease in catalytic activity of CoMn_2_O_4_ may be due to partial coverage of active sites on the surface of nanomaterials by adsorbed species, leading to pore blockage [80,81] and the leaching of Mn and Co atoms, resulting in the loss of active sites [82,83,84].

The specific surface area and pore size distribution of the catalyst after five rounds of AOPs were obtained using N_2_ adsorption–desorption isotherms (Figure 11b). Nitrogen physical adsorption analysis revealed that the specific surface area of the catalyst was reduced to about 6.41327 m^2^/g, and the pore volume was 0.01843 cm^3^/g. The hysteresis phenomenon was still presented, and the size of these holes was reduced to about 18.6 nm. This result confirmed the conjecture of repeated experiments.

## 3. Materials and Methods

### 3.1. Chemicals

Cobalt nitrate hexahydrate (Co(NO_3_)_2_·6H_2_O), manganese acetate tetrahydrate (Mn(CH_3_COO)_2_·4H_2_O), phenol (PH), benzoquinone (BQ), tert-butanol (TBA), and norfloxacin (FPA) were purchased from Shanghai MacLean Biochemical Technology Co., Ltd. (Shanghai, China). Oxalic acid (H_2_C_2_O_4_) and 10% sodium hypochlorite (NaClO) were obtained from Tianjin Tianli Chemical Reagent Co., Ltd. (Tianjin, China). Absolute ethanol, methylene blue (MB), and rhodamine B (RB) were purchased from Sinophaceutical Chemical Reagent Co., Ltd. (Beijing, China). Tetracycline hydrochloride (TC) was purchased from Shanghai Yuanyuan Biotechnology Co., Ltd. (Shanghai, China). Deionized water was used for all experiments except for studying different water matrices.

### 3.2. Synthesis of CoMn_2_O_4_

The CoMn_2_O_4_ spinel was prepared using a coprecipitation pyrolysis method. Firstly, 20 mL anhydrous ethanol solutions of 0.5 M cobalt nitrate hexahydrate and 1 M manganese acetate tetrahydrate were mixed in a conical flask with vigorous stirring at 60 °C in a water bath until completely dissolved. After addition of 150 mL of 0.24 M oxalic acid, the mixture was stirred for an additional 40 min at 60 °C. The mixture was then separated by centrifugalizing, and the obtained precipitates were washed alternately with anhydrous ethanol and deionized water three times, followed by drying the gained solid at 60 °C for 24 h. The dried solid was further calcined in a muffle furnace at 600 °C with a heating rate of 1 °C min^−1^ and maintained for 3 h. Finally, after cooling to room temperature, the obtained materials were ground and sieved through a 40–60 mesh screen for future use.

### 3.3. Experimental Procedures

Batch experiments of MB decolorization were conducted in a 1 L beaker filled with MB with a desired concentration. A default amount of NaClO and the prepared CoMn_2_O_4_ were added to the beaker while stirring on a magnetic stirrer at 500 rpm and room temperature. At predetermined time intervals, samples were withdrawn and filtered through a 0.45 μm membrane for further analysis. The initial pH of MB solution was regulated with diluted H_2_SO_4_ and NaOH if needed.

### 3.4. Characterization

The thermal analysis was studied using a SDT Q600 V20.9 Build 20 module DSC-TGA standard equipment (TA Instruments, New Castle, DE, USA) at a heating-temperature-ramping rate of 10 °C min^−1^ in the temperature range of 20–800 °C under air atmosphere. The specific surface area and porosity were determined using Micromeritics ASAP 2460 automatic (Micromeritics Instrument Corporation, Norcross, GA, USA) specific surface area and porosity analyzer (USA). The X-ray Diffraction (XRD) analysis was performed using a Rigaku Smart Lab SE diffractometer (Rigaku Corporation, Tokyo, Japan). The Fourier transform infrared spectroscopy (FT-IR) analysis was conducted using a Thermo Scientific Nicolet 6700 spectrometer (Thermo Fisher Scientific, Waltham, MA, USA). The scanning electron microscopy (SEM) was performed using a TESCAN MIRA LMS microscope (TESCAN, Brno, Czech Republic). The transmission electron microscopy (TEM) was conducted using an FEI Talos F200× microscope (Thermo Fisher Scientific, Waltham, MA, USA). The X-ray photoelectron spectroscopy (XPS) was carried out using a Thermo Scientific K-Alpha XPS instrument (Thermo Fisher Scientific, Waltham, MA, USA).

### 3.5. Analytic Methods

MB were measured using a UV–vis spectrophotometer (UV 2600) at 662 nm. The leached Mn and Co were determined using an atomic absorption spectrometer (AA-6880). The mass spectrometry analysis was conducted using an Agilent QTOF 6550 (Agilent Technologies, Inc., Santa Clara, CA, USA) with a flow rate of 0.3 mL min^−1^. The mobile phase consisted of deionized water (A) and methanol (B) in a 95:5 ratio. The mass spectrometer operated in positive ion mode with a 5 μL injection volume. Solution pH was measured using a LEICI PHS-3C pH meter (Shanghai INESA Scientific Instrument Co., Ltd., Shanghai, China).

### 3.6. Repeat Discoloration Experiment Procedures

After AOPs, the suspension was filtered to obtain catalyst powders. Then, the powders were washed alternately with anhydrous ethanol and deionized water by centrifugation three times, followed by drying the gained solid at 60 °C for 24 h. The dried catalysts were put into the reaction again, whose progress was the same as the first experimental procedure until it was circulated five times. The specific surface area and porosity of the dried powder after the five rounds of cyclic reaction were determined using Micromeritics ASAP 2460 automatic specific surface area and porosity analyzer (Norcross, GA, USA).

## 4. Conclusions

Nano-spinel CoMn_2_O_4_ was successfully synthesized using a coprecipitation pyrolysis method and confirmed via a series of characterizations. The CoMn_2_O_4_ exhibited superior catalytic activity for NaClO activation than metal oxides with an MB decolorization efficiency of 98.7% within 40 min. Under optimal conditions at a CoMn_2_O_4_ dosage of 1 g/L, an effective chlorine dose of 0.1%, and an initial pH of 4.3, the CoMn_2_O_4_/NaClO system was effective in the oxidation of various pollutants with removals higher than 90.7%. O_2_^•−^ was identified and confirmed to be the main active species for MB degradation. The nano-spinel can be reused for five cycles with slight loss of catalytic activity (less than 10%). This work provided a theoretical reference for the oxidative degradation of organic pollutants using heterogeneous activation of commercial NaClO by nano-CoMn_2_O_4_ spinel.

## Figures and Tables

**Table 1:** Degradation of MB by different catalysts and NaClO.

Catalyst	MB	Catalyst Dosage	Effective Chlorine	Time	Degradation Rate	Reference
-	1.6 mg/L	-	0.003%	80 min	85.2%	[66]
MnO_2_-MCP	100.0 mg/L	50 g/L	0.014%	300 min	98.5%	[13]
CoMn_2_O_4_	10.0 mg/L	1 g/L	0.050%	40 min	98.7%	This article

## Data Availability

The datasets used and analyzed in this study are available from the corresponding author upon reasonable request.
